# The Relationship between Environmental and Economic Aspects for Measuring the Sustainability of the Enterprise: A Case Study of Slovak Manufacturing Enterprises

**DOI:** 10.3390/ijerph19137784

**Published:** 2022-06-24

**Authors:** Emese Tokarcikova, Alzbeta Kucharcikova, Patricia Janosova

**Affiliations:** Faculty of Management Science and Informatics, Department of Macro and Microeconomics, University of Žilina, Univerzitna 8215/1, 010 26 Žilina, Slovakia; alzbeta.kucharcikova@fri.uniza.sk (A.K.); patricia.janosova@fri.uniza.sk (P.J.)

**Keywords:** sustainable development, environmental performance, carbon monoxide emissions, environmental ratio, sustainability goals

## Abstract

Despite the unbounded and undeniable advantages of manufacturing, affiliated negative externalities, such as environmental pollution, cannot be overlooked. Our article aims to focus on the current interdependence between the selected economic and environmental aspects in related manufacturing enterprises in Slovakia. We focused on analysing the relationship between carbon monoxide emissions from the largest polluters of the Slovak Republic and relevant sales. The data were taken from 83 enterprises from the Slovak Republic. Environmental and economic data were comprehensive during 2014–2019; therefore, this paper focuses on this period. Among the substantial results, we identified that carbon monoxide production from Slovak production companies was almost unchanged from 2014 to 2019, with only minimal deviations. Based on the results, we created an environmental ratio indicator as an appropriate tool for managers for their decision-making process to achieve the enterprise’s sustainability goals.

## 1. Introduction

Industrialization may have enabled how to maximize profit and increase economic prosperity, but it has also resulted in large-scale exigencies and negative impacts on the environment. On the other hand, the partial least-squares structural equation modelling technical analysis showed that environmental SDGs (0.196) had a positive influence on economic SDGs [1]. The theoretical and empirical knowledge on the research area and appropriate analysis of existing local data could improve our understanding around how manufacturing enterprises’ management can sustainably solve severe downside problems. Therefore, accumulative needs for environmental sustainability focus on reducing the negative consequences of industrial production.

We focus on understanding the relations between the financial progress of the enterprise and the related emissions. It seems to be linear, but is it? We are convinced that managers need the tools to interpret accurate data correctly to achieve sustainable results.

**Figure 1:**
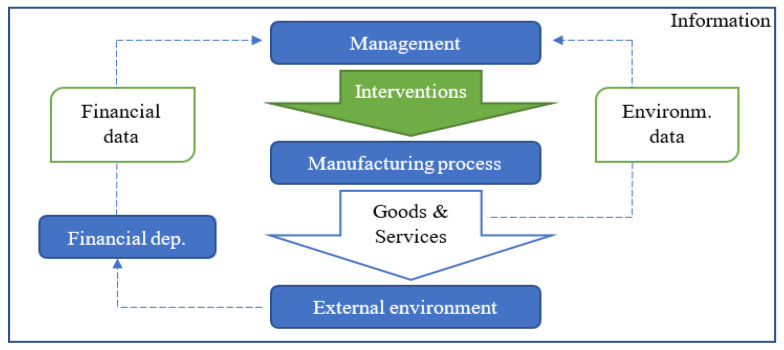
The process of obtaining and evaluating information in a manufacturing enterprise. Source: own processing.

Our initial view emerges (Figure 1) from the logical basement that management has a decision-making and control function in the enterprise. The internal and external environment of the enterprise can significantly influence its decisions. Therefore, adequately provided and formulated data obtained by the enterprises’ management must be evaluated to benefit the management. Management has all the data and information that the enterprise has at its disposal. There is still uncertainly around how to use these data and information correctly (interventions).

The rapid expansion of industrial production in the second half of the 20th century significantly affected the Slovak economy and other post-communist countries. The number of industrial enterprises increased the importance of industry, including engineering, metalworking, and chemical industries. However, as demonstrated by Haggard and Kaufman (2008), a stronger emphasis on the development of industry in post-communist states also brought shadowy effects on environmental pollution, which were not given considerable attention [2]. Nevertheless, sustainability efforts have changed over the years. Emphasis is now placed on the prosperity of the country and its economic aspect, as well as the lives of people and the environment. This breakthrough brings significant social and environment changes as a necessary part of the activity of every enterprise. Many enterprises build a solid relationship with their stakeholders through corporate social activities and build a strong commitment to being environmentally friendly.

The paper is organized as follows. In Section 1, we present a literature review mainly on industry and environmental relations issues. In Section 2, the methodology and some hypotheses are presented. In Section 3, we present the main results and a discussion about the main empirical findings. Finally, we offer concluding remarks.

## 2. Literature Review

Manufacturing enterprises are the pillars of each economy. As stated in manufacturing statistics from Eurostat and Herman’s (2016) research results, the manufacturing sector generates economic activities based on products demanded by production (transformation of input into the output); job creation for the workforce (a reduction in unemployment); and the ability for multiplier effects to contribute to GDP, living standards, and the whole future of economics [3,4]. Despite these clear benefits, striving for profit maximization (regardless of the consequences that may negatively affect the quality of the enterprise’s surroundings, stakeholders, and environment) generated a lot of social and environmental costs.

Convergence approaches to modelling relation among per capita income and emissions of various pollutants, such as carbon dioxide, nitrogen oxides, and other various indicators of environmental degradation, primarily use the environmental Kuznets curve (EKC). The hypothesis of the EKC is based on the idea that economic prosperity initially leads to a deterioration in the environment, but after a certain level of economic growth, society can improve and stop environmental degradation [5].

The results of Zortuk and Ceke demonstrate there is an apparent non-linear relationship between CO_2_ emissions per capita and GDP per capita in the selected eleven transition economies from 1993 to 2010. “However, considering additional variables that may affect the dependent variable could enable more accurate results for further studies” [6]. Makreshanska-Mladenovska and Petrevsi (2019) also say that for a panel of 11 economies from Central and Eastern Europe (CEE) as former communistic economies, they cannot confirm the validity of the Kuznets hypothesis [7]. Pilatowska and Wlodarczyk (2017) also identified a piece of significant evidence that EKC holds between per capita CO2 and GDP per capita for Slovakia, Romania, and the Czech Republic caused by the effective environmental policy of these countries [8]. In a related research study, Stern argues that evidence for the inverted U-shaped curve of the EKC applies only to a subset of environmental measures, and it must improve. According to findings, understanding and identifying the factors that are non-growth drivers of pollution reduction is essential [9,10]. Shah et al. quantified the EKC validity against the ecological footprint and found the alternative one more valid [11].

These undoubted facts on the relation of economic prosperity and environmental damage have forced enterprises, governments, and significant international authorities to focus on worldwide reflection regarding human activities, business processes, and manufacturing impact on the future of the environment and humankind. The Brundtland Report in the 1980s defined sustainability development as the “developments that meet the needs of the present without compromising the ability of future generations to meet their own needs” [12]. Following that, many researcher studies declare [13,14,15] the necessity to transform a united approach into all levels of society and its organizations of different kinds to implement Agenda 2030 and to contribute to the consolidation of the Sustainable Development Goals (SDGs). As mindsets change, we recognise that key manufacturing enterprises which span across several industries, including electronics, automotive, food and beverage, chemicals, pharmaceutical and medical equipment, among others, aim toa chieve green innovation, sustainability, and agility. Following that, other sectors implemented change—for example, researchers declared new sustainable transportation modes [16,17,18] or well-focused investments through green business innovations [19,20,21]. In general, empirical studies based on Lorincova et al. (2019) and Raisiene et al. (2020) argue that stakeholders’ motivation is critical for achieving sustainability within business processes [22,23]. Certain priorities to find effective managerial and economic methods and measures with reasonable attitude include the education and motivation of managers and employees, and research studies highlight the proper usage of technological innovations and software solutions [24,25,26,27].

Indeed, in manufacturing enterprises, Industry 4.0, whose essence is the use of technology for efficient production, offers massive potential to create support that guarantees higher environmental protection and sustainability with more positive impact than before [28]. Despite the high implementation costs of technologies, such as AI, the Internet of Things, advanced data analytics, robotic process automation, blockchain, robotics, cloud computing, virtual and augmented reality, 3D printing and drones, and 5G (as it continues to roll out), they offer environmentally friendly solutions, new business models, competitive advantage [29] and sustainable value creation, innovation, and investments in all sustainability dimensions [30]. From the 1960s onwards, environmental problems began to emerge, which several countries began to address [31]. The current state of environmental pollution is unsustainable, and many organizations are aware of it [32]. Emissions are aggregated to five anthropogenic sectors: power, industry, residential, transportation, and agriculture [33]. In recent years, carbon neutrality has received considerable attention, mainly in the European Union countries, as the European Union is one of the third largest producers of greenhouse gases [34].

### Emissions as an Associated Product of Enterprises—Examination of Environmental Performance

Environmental quality is considered an important asset, especially in developed countries [35]. The relationship that addresses the link between an enterprise’s financial performance and its environmental burden has been of great interest in recent years, especially among researchers and managers [36]. It also has application in terms of demand from stakeholders, especially when deciding on their investments [37]. Despite the recurring frequency of use, the exact definition of environmental performance is not precisely defined in the literature. However, we can define it as “a measure of effort that compares the economic and environmental indicators of an enterprise” [37].

Several studies in the literature assess the relationship between economic and environmental aspects [38]. However, it is also necessary to grasp this issue at a practical level, which will provide business managers with a framework suitable for assessing the degree of sustainable development. Several studies that analyse the relationship between financial and environmental aspects focus only on one-way causality, which is specified in more detail in the studies by Muhammad et al. (2015) and Qian (2012) [39,40]. More specifically, it is a unilateral examination of how environmental policy affects financial policy. There is still a lack of research on the impact of fiscal policy on environmental policy. Measuring and recording performance is a very demanding process, which requires a unique approach in each enterprise [41]. Several studies focus on examining specific environmental performance. These include, e.g., research by Rios and PicaZo-Tadeo (2021), who addressed the European Union’s environmental performance in solid waste [42], or research by Hospido et al. (2004), which deals with environmental performance in the field of wastewater [43]. In addressing the issue of environmental performance in emissions in scientific databases, studies dealing exclusively with greenhouse gases predominate, e.g., those by Abban and Hasan (2021), Earnhart and Lizal (2010), and Quian and Xing (2019) [44,45,46]. However, studies on other pollutants are lacking. The most pollutants by U.S. Environmental Protection Agency that need to be reduced include PM 2.5 and PM 10 particulate matter; carbon dioxide; nitrogen oxides; sulphur oxides; and, finally, carbon monoxide, which is an essential factor in our paper [47]. Carbon monoxide is perceived as a significant factor that continuously contaminates the Earth’s atmosphere and has fatal effects on living organisms [48]. Several studies which aimed to reduce carbon monoxide (CO) agree that CO values can be reduced mainly through technical principles. Researchers in various parts of the world have conducted research (e.g., Ulcak and Kassouri, 2020; Feist, et al., 2020; and Rehman, et al., 2020) to address the interaction between carbon dioxide and economic progress. In conclusion, it was found that strict environmental regulatory policies, which include environmental taxes, are not sufficient to reduce carbon values [49,50,51]. An internal initiative of each participating element in emission production is required, based on its own beliefs. In this paper’s case, we refer to the internal conviction of the management of each enterprise, which should consider the importance and necessity of its decisions. The analysis, evaluation, optimization, and control of these measures should be an essential part of this process. These activities represent a continuous process to reduce the negative impact of the enterprise on the environment. Useful practical implications for managers, including a study by Essid and Berland, show how organizational capabilities, dynamic and ordinary, are operationalised in the adoption of environmental management tools [52].

## 3. Materials and Methodology

In this paper, we focus mainly on the analysis of the relationship between economic and environmental indicators—more precisely, the relationship between the enterprise’s revenues and carbon monoxide emissions.

**ERIco is an environmental ratio indicator of carbon monoxide** created by us. It compares the selected economic and environmental indicators. Initial economic data describe Slovakian manufacturing companies’ revenue comes from the sale of the own products and services from 2014 to 2019 (cross control through balance sheets, profit and loss statements, and data from the Slovakian financial website (www.financial.sk, accessed on 12 December 2021)). Revenues from the sale of its own products and services were chosen based on a high informative value of the companies’ financial situation. We selected the emissions due to the high proportion of air pollution in the Slovak Republic, as well as public reports and recordings. These environmental data from all analysed companies were gained from the statistics of the Slovak Hydrometeorological Institute. Indicator ERIco can take various forms, depending on the data we have. In this case, we calculated the ratio indicator’s value as the ratio of the amount of carbon monoxide emissions and its revenue from the sale of the own products and services for a given period. For the sake of better clarity, we divided the result by the number 1,000,000. This number depends on the range of revenues that the analysed enterprise receives.

Before collecting the financial data, themselves, and the value of the pollutants, we focused on answering the following research questions. These research questions focus on the need to deal with metrics that contain environmental and economic data intended for business management.

Q1: What are the possibilities for reducing CO emissions from the management’s point of view?

Q2: How many CO emissions does the average enterprise produce in the case of EUR 1 sales revenue?

Q3: What is the trend in the amount of CO emissions produced in relation to the amount of revenues generated by enterprises?

To answer our paper’s underlying research question, we applied the following hypotheses and verified the truth value. The hypotheses are focused on ERI_CO_ testing.

**Hypotheses 1 (H1):**  *The average value of ERI_CO_ in 2019 was higher than in 2014. (If this hypothesis is confirmed, we conclude that it is true, it means that enterprises in 2019 produce higher revenues with lower emissions than in 2014).*

**Hypotheses 2 (H2):**  *In 2019, enterprises produced on average fewer emissions with EUR 1 sales than in 2014. (If this hypothesis is confirmed, we conclude that it is true, it means that enterprises in 2019 received higher sales at the same emissions in 2014).*

**Hypotheses 3 (H3):**  *The mean value of the carbon monoxide indicator in 2019 and 2014 was the same. (If this hypothesis is confirmed, we can state that environmental measures in enterprises are constant).*

These hypotheses were verified by one-way analysis of variance (ANOVA). The significance level was set at 0.05 in both cases. For better clarity, we collected data from 83 manufacturing enterprises in the Slovak Republic into the relevant regions of Banskobystrický (BB), Bratislavský (BA), Košický (KE), Nitriansky (NR), Prešovský (PO), Trenčiansky (TN), Trnavský (TT), and Žilinský (ZA).

The number of groups of ERI_CO_ values is formed based on the relationship:

k = 1 + 3.3logn,(1)

n is the number of elements.

The values of the ERI_CO_ value intervals are formed based on the relationship:

h = (x_max_ − x_min_)/k (2)

x_max_ is the highest achieved ERI_CO_ value

x_min_ is the lowest achieved ERI_CO_ value.

The ERI_CO_ value intervals can be created for each year separately. The reason is the emergence of a scale from the best result to the worst in each year. If the same intervals were created for all analysed years, the result would be a trend of ERI_CO_ development within years. Values acquired during older years would not provide a relevant indicative value compared to subsequent years.

## 4. Results and Discussion

After calculating the basic characteristics, we found that the ERI_CO_ takes values from 0 to 1.678. In general, we can say that the higher the value, the more sustainable the enterprise—the emissions produced are lower in terms of sales. Based on the relationships defined in the methodology, the result is 3.9802 interval groups.

The resulting ERI_CO_ values are shown in Table 1. The difference between the best ERI_CO_ values between the first and the last analysed year is 1.1915, representing an increase of 239.69%. The highest value was recorded in 2019 in the Bratislava Region (1.69) and the lowest in 2015 in the Košice Region (0.02). The difference between the worst ERI_CO_ values is 0.022, i.e., 92.611% in percentage terms. In 2019, compared to 2014, much fewer emissions were eliminated due to corporate revenues. Most of the data listed in Table 1 belong to the “black zone”. These data show that many enterprises do not sufficiently reduce their carbon monoxide emissions compared to the achieved revenues volume.

**Figure 2:**
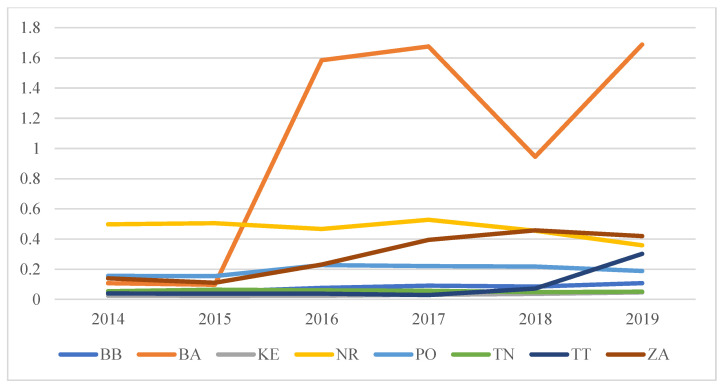
Graphical evaluation of ERI_CO_ values during the years 2014–2019. Own processing.

**Figure 3:**
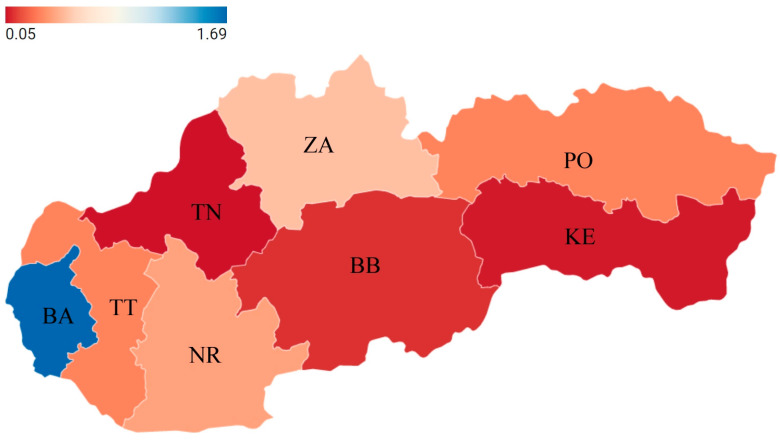
Overview of ERI_CO_ values in the regions of the Slovak Republic during 2019. Own processing.

The following Figure 2 provides a graphical representation of the situation and clear development of the ERI_CO_. It shows that almost all regions maintain the ERI_CO_ value in the range of 0–0.6. Only the Bratislava Region (Figure 3) differs significantly (the best) in ERI_CO_ values for the selected period.

The values from 2016 to 2019 are positive. This result means that enterprises in this region can increase their revenues by decreasing, especially in the case of constantly produced carbon monoxide emissions.

To determine the truth value of H1, it is necessary to compile Table 2, which contains the average ERI_CO_ values. A graphical representation of the situation with the forecast for future years is contained in Figure 3, which compares the actual average ERI_CO_ values with the average ERI_CO_ values after excluding the outliers.

This map (Figure 3) is important because it shows where there are significant differences in the value of the ERIco indicator in individual regions of the Slovak Republic. The highest values are acquired by the Bratislava Region, which is important because based on enterprises in the Bratislava Region, in a future study, the best practices (especially objectives, practices, and its implementation) of these enterprises could be performed for use in other enterprises in the Slovak Republic.

The situation that has arisen shows that the use of real data is growing, which is beneficial for the country and beneficial for sustainable direction. On the other hand, after removing the outliers, the resulting trend has a declining character. The paradox of these trending lines is that the growing development of ERI_CO_ values is due to the high values of ERI_CO_, originating from the Bratislava region.

Hypothesis H1 can be accepted and considered valid if we consider all the data. Otherwise, after excluding outliers, Hypothesis H1 can be rejected.

**Figure 4:**
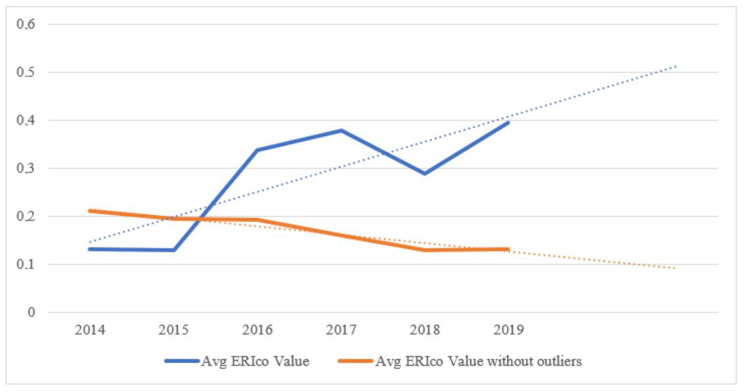
Forecast of ERI_CO_ of actual values and ERI_CO_ after exclusion of outliers. Own processing.

Even in this case (Figure 4), it is necessary to draw attention to the fact that the positive trend and the forecast of the values of the ratio indicator are significantly affected by the positive values acquired by the Bratislava Region for a long time.

To evaluate the H2 hypothesis, it is necessary to recalculate the amount of emissions produced to the relevant financial units.

**Figure 5:**
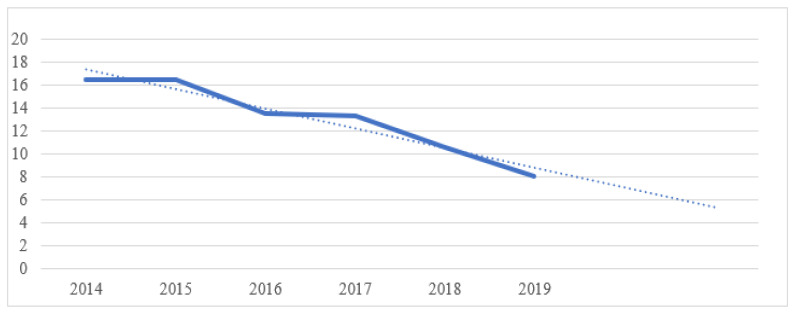
Average amount of emissions produced per monetary unit. Own processing.

Figure 5 shows that the average productivity of CO emissions to achieve EUR 1 of sales is declining every year. In 2014, an average of 16.49 g of CO emissions were produced to achieve sales of EUR 1, while only 8.098 g of CO emissions were produced in 2019. **As a result, we can confirm hypothesis H2. The reason is that demonstrably less emissions were produced to generate sales of EUR 1 in 2019 compared to 2014.**

The maximum permissible values for the level of carbon monoxide in the air according to the National Institute for Occupational Safety and Health (1978) are 55 mg/m^3^. However, the recalculation of the permissible value of CO produced by Slovak enterprises in spatial terms is not stated in this paper.

There is still conflict among researchers on the appropriateness and adequacy of measures that could bring increasing profits to businesses, but the rate of adverse environmental impacts will decrease. These impacts are often not avoided without additional costs in the form of innovation. However, it is important to realize, especially from the perspective of managers, that from each profit unit in which a negative burden on the environment has arisen, it is necessary to make financial investments to compensate for the previous negative impact.

We see significant potential and an unexplored area in focusing on all substances that have an adverse impact on the environment, the impact of greenhouse gas emissions, and other pollutants. We also see the potential in examining the impact of specific sectors’ activities on the environment with defined financial indicators.

We evaluate the last hypothesis based on a paired T-test (Table 3) because individual enterprises and their emissions produced annually have a demonstrable connection. The table of evaluated data is given below.

It is clear from Table 3 that the value of the alpha parameter that we determined is 0.05 smaller than the resulting *p*-value. For this reason, we do not reject the H3 hypothesis, and we can say that the production of emissions by companies is the same during 2014 and 2019. It means that year-on-year initiatives from the state and companies’ point of view to improve the environment’s quality are not sufficiently proven.

Certainly, it is impossible to infer the company’s environmental impact by considering the emission rate of only one pollutant. Since CO emissions are practically always associated with emissions of other pollutants, the relationship between the enterprise’s revenues and greenhouse gas emissions is relevant. Our findings in this article are only a part of our complex research according to the sustainability intentions of Slovakian manufacturing companies. When assessing the degree of sustainable development in manufacturing companies, there is often a problem in correctly identifying indicators. PWC (2019) suggests that sustainable development reporting is not sufficiently developed, especially in the private sector [53]. Our findings on Slovakian conditions suggest that they do not understand them in many cases or cannot use them for decision making, as every manufacturing company has some specific production process and related emissions. ERIx is a very intuitive and flexible index for recording, understanding, and using the management decision process at any level. Yes, it is only the first step towards sustainability activities in the company, but it is an important one.

Another limitation of our calculations on the annual changes in the average productivity of CO emissions to achieve EUR 1 of sales is that we did not take into account the inflation rate, which is nowadays a significant parameter used to improve forecasting of ERI_CO_ values, but also proper usage and interpretation of values of general index ERIx.

There are also no known recommendations identifying adequate tools for the benefits of sustainable business development. In their study, Di Vaio and Varriale (2020) draw attention to the fact that many manufacturing manufacturers lack guidelines and frameworks as a guide for management application [54]. The sustainable development report has a dual meaning for the company. First, it is a communication tool (for stakeholders) which reflects the activities performed in the company; on the other hand, it measures the manufacturing company’s progress in sustainable development and identifies new strategies and goals in the context of sustainable development. Pavlik and Belcik (2010) recommend evaluating the sustainable development report based on the following criteria:

- Completeness of information in the report, which means informing about all facts supporting the sustainable development of the company, including changes in indicators that have occurred;
- Materiality in the sense of avoiding a vague description and a correctly chosen indicator for assessing sustainable development in the company;
- Credibility is created by the approval of the report by the company’s top management as well as the company’s stakeholders;
- A report form that is transparent and easily processed graphically [55].

## 5. Conclusions

Since the end of the 20th century, the amount of emissions produced into the air has been an increasingly discussed topic in the scientific community and among essential representatives of the United Nations and the European Union. Emissions to air harm human, animal, and all living organisms. It is also essential to address health issues from a macroeconomic and microeconomic perspective [56,57]. Exploring the relationship between an enterprise’s financial and environmental aspects is a much discussed topic among members of the scientific community and managers in enterprises. We know that every business’s main goals are prosperity and profit-making [58], but these must not be sought at the expense of the environment’s quality. Several studies that have been carried out in this area focus on environmental performance only unilaterally, or in most cases, deal with the production of carbon dioxide because we classify this gas as a greenhouse gas. It is important to note and address other pollutants as well.

This indicator is easily usable in all manufacturing companies that record the production of emissions during the production process. We analysed data of 100 Slovak companies, which are among the country’s most critical air pollutants, and identified the relationship between the company’s revenues as a financial value and carbon monoxide emissions as an environmental value. As there is a demonstrable link between these two indicators, we consider it necessary to deal with them in more detail because managers can take advantage of them for further development of sustainability activities in the company. It also serves them for precise continuous control of the company’s environmental impact. We showed a forecast trend and drew attention to the fact that the positive trend and the forecast of the values of the ratio indicator are significantly affected by the positive values acquired by the Bratislava Region for a long time. Additionally, we can confirm that after our verification dialogues with the manufacturing companies’ managers, the first three companies monitored and reported the general index ERIx into their processes based on the nature of their production process and the extent of its environmental impact. One company also required a carbon footprint reporting an option synergy with ERIx.

## Figures and Tables

**Table 1:** ERI_CO_ values during 2014–2019.

	2019	2018	2017
*BB*	0.107498598	0.084806818	0.090367001
*BA*	1.688662866	0.945114517	1.675673504
*KE*	0.046246007	0.03596897	0.029705687
*NR*	0.35847519	0.454249924	0.527491725
*PO*	0.187615212	0.218155193	0.220685604
*TN*	0.051410005	0.047785933	0.057088947
*TT*	0.301658593	0.071379045	0.028811798
*ZA*	0.419680074	0.457426389	0.394163716
	**2016**	**2015**	**2014**
*BB*	0.07650958	0.051454776	0.047895658
*BA*	1.5844525	0.096681365	0.106422525
*KE*	0.024639643	0.023502785	0.02401011
*NR*	0.466223316	0.505207176	0.497117964
*PO*	0.227271689	0.154435191	0.155544138
*TN*	0.060483756	0.064229745	0.053683627
*TT*	0.037362492	0.037324523	0.038862762
*ZA*	0.231390935	0.110232727	0.138972132

Own processing.

**Table 2:** Average ERI_CO_ values in the Slovak Republic in the period 2014–2019.

	2014	2015	2016	2017	2018	2019
**Avg ERI Value**	0.132813	0.130384	0.338542	0.377998	0.289361	0.395156

Own processing.

**Table 3:** Results of using two paired samples in the means T-test for H3 verification.

PARAMETERS	CARBON MONOXIDE (2019)	CARBON MONOXIDE (2014)
**MEAN**	1178.69	1729.3
**VARIANCE**	54,426,689.05	155,735,223.2
**OBSERVATIONS**	83	83
**PEARSON CORRELATION**	0.99	0.99
**HYPOTHESIZED MEAN DIFFERENCE**	0	0
**DF**	82	82
**T STAT**	−0.96	−0.96
**P(T ≤ T) ONE-TAIL**	**0.17**	**0.17**
**T CRITICAL ONE-TAIL**	1.66	1.66
**P(T ≤ T) TWO-TAIL**	0.34	0.34
**T CRITICAL TWO-TAIL**	1.99	1.99

## Data Availability

Some data generated or analysed during this study are included in this published article. The whole datasets generated during and/or analysed during the current study are available from the corresponding author on reasonable request.
